# Genome-Wide Association Analysis of Oxidative Stress Resistance in *Drosophila melanogaster*

**DOI:** 10.1371/journal.pone.0034745

**Published:** 2012-04-04

**Authors:** Allison L. Weber, George F. Khan, Michael M. Magwire, Crystal L. Tabor, Trudy F. C. Mackay, Robert R. H. Anholt

**Affiliations:** 1 Department of Genetics, North Carolina State University, Raleigh, North Carolina, United States of America; 2 W.M. Keck Center for Behavioral Biology, North Carolina State University, Raleigh, North Carolina, United States of America; 3 Department of Biology, North Carolina State University, Raleigh, North Carolina, United States of America; Vetmeduni Vienna Institute of Population Genetics, Austria

## Abstract

**Background:**

Aerobic organisms are susceptible to damage by reactive oxygen species. Oxidative stress resistance is a quantitative trait with population variation attributable to the interplay between genetic and environmental factors. *Drosophila melanogaster* provides an ideal system to study the genetics of variation for resistance to oxidative stress.

**Methods and Findings:**

We used 167 wild-derived inbred lines of the *Drosophila* Genetic Reference Panel for a genome-wide association study of acute oxidative stress resistance to two oxidizing agents, paraquat and menadione sodium bisulfite. We found significant genetic variation for both stressors. Single nucleotide polymorphisms (SNPs) associated with variation in oxidative stress resistance were often sex-specific and agent-dependent, with a small subset common for both sexes or treatments. Associated SNPs had moderately large effects, with an inverse relationship between effect size and allele frequency. Linear models with up to 12 SNPs explained 67–79% and 56–66% of the phenotypic variance for resistance to paraquat and menadione sodium bisulfite, respectively. Many genes implicated were novel with no known role in oxidative stress resistance. Bioinformatics analyses revealed a cellular network comprising DNA metabolism and neuronal development, consistent with targets of oxidative stress-inducing agents. We confirmed associations of seven candidate genes associated with natural variation in oxidative stress resistance through mutational analysis.

**Conclusions:**

We identified novel candidate genes associated with variation in resistance to oxidative stress that have context-dependent effects. These results form the basis for future translational studies to identify oxidative stress susceptibility/resistance genes that are evolutionary conserved and might play a role in human disease.

## Introduction

Oxidative stress, or the overabundance of reactive oxygen species (ROS) as an unavoidable consequence of aerobic respiration, has been implicated in aging [1], [2], neurodegenerative and cardiovascular disease [3], [4] and the disruption of cell signaling processes that control cell growth and death [5]. Excessive accumulation of ROS can damage DNA and proteins and disrupt critical cellular signaling pathways, which ultimately leads to the breakdown of cellular processes and contributes to organismal aging and disease susceptibility [6], [7]. Oxidative stress may contribute to dysfunction and death of neuronal cells that occur in Alzheimer's disease, Parkinson's disease and amyotrophic lateral sclerosis [3]. ROS may also induce cardiovascular disease by affecting signaling pathways that lead to inflammation of vascular tissue in atherogenesis, the process where fat deposits form on the inner lining of blood vessels [8], [9], [10].

Genetic variants associated with susceptibility to oxidative stress in human populations have largely been identified indirectly, as many alleles associated with increased risk for common diseases and aging are in genes in oxidative stress response pathways [4]. Model organisms offer the advantage of direct screens for variants affecting survival following exposure to acute oxidative stress inducing agents. Alleles of *age-1*, *mth*, and *shc^66^* associated with increased longevity of *Caenorhabditis elegans*, *Drosophila melanogaster* and mice [11], [12], [13], [14], respectively, have pleiotropic effects on increased resistance to oxidative stress. Biochemical studies have implicated *Superoxide dismutase* (*Sod*) [15], [16] and *Catalase* (*Cat*) [17], [18] as fundamental mediators for the removal of ROS. Overexpression of both *Sod* and *Cat* transgenes results in enhanced oxidative stress resistance and longevity in specific *Drosophila* genetic backgrounds [19], [20], [21]. Studies characterizing changes in genome-wide gene expression following induction of oxidative stress implicate a large number of genes, including genes associated with purine biosynthesis and innate immune response [22]. Genome-wide expression has also been assayed on lines that have undergone multigenerational selection for increased resistance to hyperoxia. This resulted in the identification of several candidate genes involved in survival under hyperoxia, including *Diptericin*, *Tropomyosin1*, and *Attacin* [23]. Genome-wide transcriptional profiling revealed that different methods of inducing oxidative stress elicit a few common transcriptional responses, but the majority of gene expression changes are specific to the oxidative stress inducing agent [24]. Naturally occurring genetic variation for oxidative stress resistance has been identified by mapping quantitative trait loci (QTLs) in recombinant inbred lines (RILs) of *D. melanogaster* [25], [26]. However, the sampling of natural genetic variation in these studies was limited, since the RILs were generated from only two wild-caught female flies [27].

Recently, a population of inbred wild-derived lines comprising the *D. melanogaster* Genetic Reference Panel (DGRP), derived from the Raleigh, North Carolina population, has been fully sequenced [28]. The DGRP affords the opportunity for the first time to perform genome-wide association (GWA) analyses for quantitative traits when all genetic variants are known. The low level of local linkage disequilibrium (LD) in the DGRP is favorable for identifying candidate causal polymorphisms. The DGRP lines are inbred, which enables rearing nearly genetically identical individuals in large numbers, thereby increasing the power to reduce statistical noise due to environmental variation. Furthermore, inbreeding of the DGRP lines has minimized residual heterozygosity and increased the genetic variance among lines to at least double that of the outbred population from which it was derived [29], which also increases power for GWA mapping. The *Drosophila* model allows validation of candidate genes and identification of causal SNPs through a variety of genetic approaches, including analysis of mutations.

Here, we have used 167 DGRP lines to conduct GWA mapping of acute oxidative stress susceptibility/resistance by measuring survival time on two different oxidative stress inducing agents, paraquat and menadione sodium bisulfite (MSB). We found significant and sex-specific genetic variation in survival time among the lines for both treatments. Our GWA analysis identified several hundred candidate genes, several of which were previously not known to be involved in oxidative stress response and many of which have human homologs. Many of these candidate genes were sex-specific and uniquely susceptible to one of the oxidative stress inducing treatments, but a subset converge on a common network that may represent a central conduit for response to various oxidative stress inducing agents. Finally, we have validated a subset of candidate genes using mutant analysis.

## Results

### Natural variation in oxidative stress susceptibility

**Figure 1:**
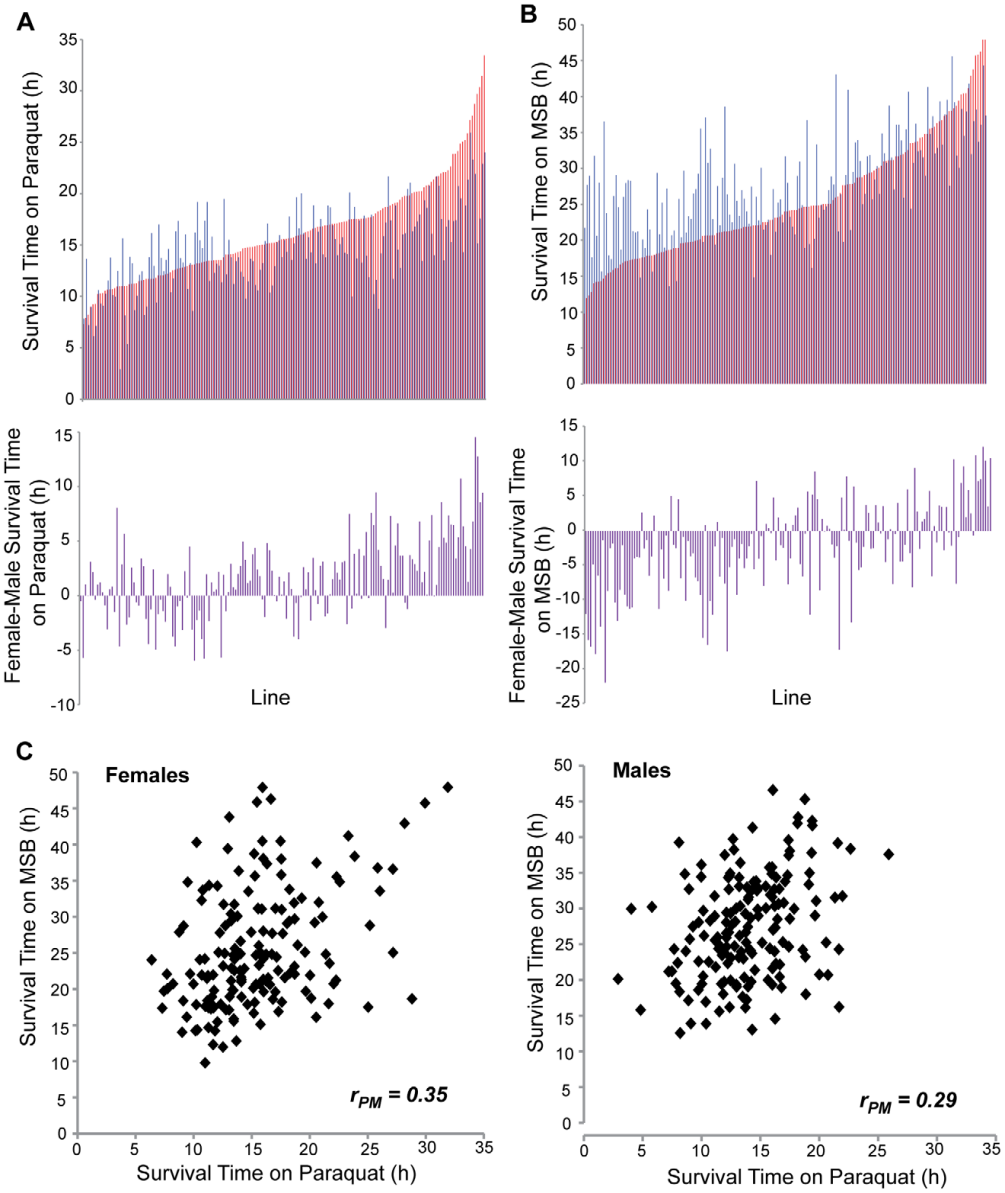
Variation in oxidative stress resistance among 167 DGRP lines. Line means for survival time on paraquat (A) and MSB (B) for females (red bars) and males (blue bars), and sexual dimorphism (female-male) (purple bars). (C) Genetic correlation of survival times on paraquat and MSB (*P*<0.0001) for females and males separately. Mean phenotypic values for each line-sex are given in Table S1.

To characterize natural genetic variation in oxidative stress susceptibility, we measured survival time on two different oxidative stress inducing agents, paraquat (1,1′-dimethyl-4,4′-bipyridinium dichloride) and menadione sodium bisulfite (MSB), among 167 DGRP lines [28], for males and females separately (Figure 1A–B; Table S1). The DGRP lines vary in *Wolbachia* infection status [28]. *Wolbachia* infection had a significant effect on survival time on paraquat for both sexes and survival time on MSB in males (Table S2). Therefore, we adjusted the phenotypic values according to *Wolbachia* infection status for subsequent analysis.

We found extensive phenotypic and genetic variation for survival time on both paraquat and MSB (Figure 1A–B; Table 1), similar to the broad variation observed for other traits in this population [28], [30]. The broad sense heritability (*H^2^*) for survival time was *H^2^* = 0.48 on MSB and *H^2^* = 0.36 on paraquat (Table 2), when pooled across sexes. Survival on exposure to both paraquat and MSB is sexually dimorphic: averaged over all lines, survival of males is 90% that of females on paraquat, whereas survival of females is 87% that of males on MSB (Table S1). However, there was significant genetic variation in the magnitude and direction of sexual dimorphism for survival under both oxidative stress inducing agents, as indicated by the significant sex by line interaction terms in the analyses of variance (Table 1). Cross-sex genetic correlations (*r_MF_*), which quantify the extent to which the same variants affect a trait in males and females, were significantly different from unity (paraquat, *r_MF_* = 0.70; MSB, *r_MF_* = 0.64), underscoring sex-specific differences in susceptibility to oxidative stress agents.

**Table 1:** Analyses of variance of survival time on paraquat and MSB.

Trait	Analysis	Source of Variation	df	MS	*F*	*P*-value	*σ^2^*
Survival Time on Paraquat	Sexes Pooled*	Sex	1	4605.06	29.41	<0.0001	
		Line	166	726.28	4.64	<0.0001	12.38
		Sex*Line	166	156.56	5.10	<0.0001	5.25
		Error	7682	30.72			30.72
	Females*	Line	166	533.61	15.49	<0.0001	20.80
		Error	3841	34.458			34.46
	Males*	Line	166	349.23	12.94	<0.0001	13.43
		Error	3841	26.98			26.98
Survival Time on MSB	Sexes Pooled*	Sex	1	26808	48.90	<0.0001	
		Line	166	2315.15	4.22	<0.0001	36.81
		Sex*Line	166	548.20	8.83	<0.0001	20.25
		Error	7682	62.10			62.10
	Females	Line	166	1598.23	22.74	<0.0001	63.66
		Error	3841	70.28			70.28
	Males*	Line	166	1265.12	23.47	<0.0001	50.47
		Error	3841	53.91			53.91

df: degrees of freedom; MS: Type III Mean Squares; *σ^2^*: variance component.

* Phenotypic line-sex means adjusted for *Wolbachia* infection status.

**Table 2:** Quantitative genetic analysis of survival time on paraquat and MSB.

Parameter	Symbol	Survival Time on Paraquat*	Survival Time on MSB*
Mean	*μ*	14.70	27.28
Genetic variance	*σ_G_^2^*	17.63	57.06
Genetic standard deviation	*σ_G_*	4.20	7.55
Environmental variance	*σ_E_^2^*	30.72	62.10
Environmental standard deviation	*σ_E_*	5.54	7.88
Phenotypic variance	*σ_P_^2^*	48.35	119.16
Phenotypic standard deviation	*σ_P_*	6.95	10.92
Heritability	*H^2^*	0.36	0.48
Coefficient of genetic variation	*CV_G_*	28.57	27.68
Coefficient of environmental variation	*CV_E_*	37.69	28.89
Cross-sex genetic correlation	*r_MF_*	0.70	0.64

* Phenotypic line-sex means adjusted for *Wolbachia* infection status.

When sexes were assessed separately, pooled across treatments (Table S3; Table S4), the effect of treatment was significant for both males and females, with greater survival times on MSB than paraquat. Averaged over all lines, the survival time on paraquat was 61% that of survival on MSB for females; while the average survival time on paraquat was only 48% that of survival on MSB for males (Table S1). However, there was significant genetic variation in the magnitude and direction of the effect of treatment in both sexes, as indicated by the significant line by treatment interaction terms in the analyses of variance (Table S3). Genetic correlations between resistance to oxidative stress for the two treatments were significantly different from zero and similar for both sexes (female *r_PM_* = 0.35; males *r_PM_* = 0.29; Figure 1C), but not high, indicating that largely different genetic variants affect the response to acute oxidative stress induced by paraquat and MSB. Thus, the genetic architecture of oxidative stress susceptibility is sex-specific and dependent on the method of induction.

### SNPs associated with oxidative stress susceptibility

**Figure 2:**
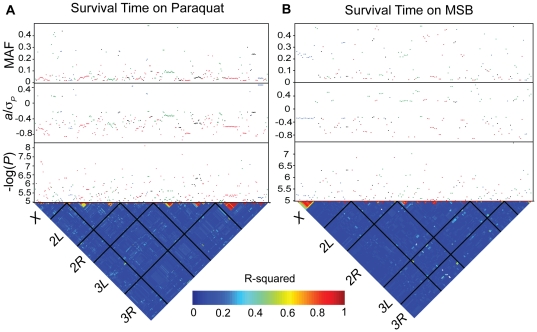
Genome-wide association analyses for survival time on paraquat and MSB (sexes pooled). All SNPs from single marker analyses with a nominal *P*<10^−5^ are shown. Associations based on females are depicted by red dots, males by blue dots, sexes pooled by black dots and SNP by sex interactions by green dots. The lower triangle depicts the degree of LD between SNPs as measured by *r^2^*, with the five major chromosome arms demarcated by the black lines. The heat map indicates the magnitude of LD with red corresponding to complete LD and blue to absence of LD. The upper panels show the significance threshold (−log_10_ *P*), the effect size in phenotypic standard deviation units (*a*/*σ_P_*), and the minor allele frequency (MAF). (A) Survival time on paraquat. (B) Survival time on MSB.

**Figure 3:**
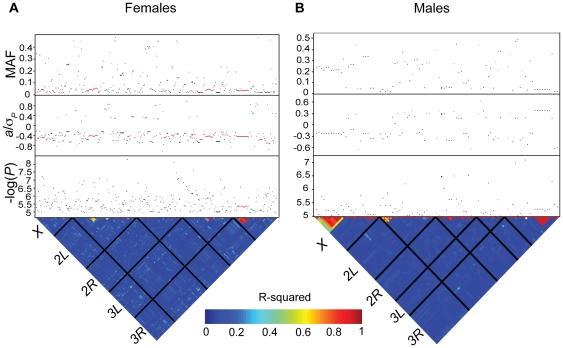
Genome-wide association analyses for females and males (treatments pooled). All SNPs from single marker analyses with a nominal *P*<10^−5^ are shown. Associations based on survival on paraquat are depicted by red dots, survival on MSB by blue dots, treatments pooled by black dots and SNP by treatment interaction by green dots. The lower triangle depicts the degree of LD between SNPs as measured by *r^2^*, with the five major chromosome arms demarcated by the black lines. The heat map indicates the magnitude of LD with red corresponding to complete LD and blue to absence of LD. The upper panels show the significance threshold (−log_10_ *P*), the effect size in phenotypic standard deviation units (*a*/*σ_P_*), and the minor allele frequency (MAF). (A) Females. (B) Males.

To identify genes that harbor alleles that confer genetic risk to paraquat- or MSB-induced oxidative stress, we performed GWA analyses for survival time on paraquat and MSB with 2,481,491 SNPs previously identified by sequencing the DGRP lines [28]. We performed single marker analyses pooled across sexes and for males and females separately within each treatment. We also performed analyses pooled across treatments for each sex. We found 298 SNPs that were associated with phenotypic variation for survival time on paraquat and 154 SNPs that were associated with phenotypic variation for survival time on MSB at *P*<10^−5^. A total of 56 (23) remained significant for paraquat (MSB) when the significance threshold was *P*<10^−6^ (Figure 2; Table S5). When we considered the sexes separately, we found 327 (92) SNPs associated with survival time on either or both oxidative stress agents for females (males) at *P*<10^−5^. A total of 69 SNPs remained significant for females and 9 for males at *P*<10^−6^ (Figure 3; Table S6).

**Figure 4:**
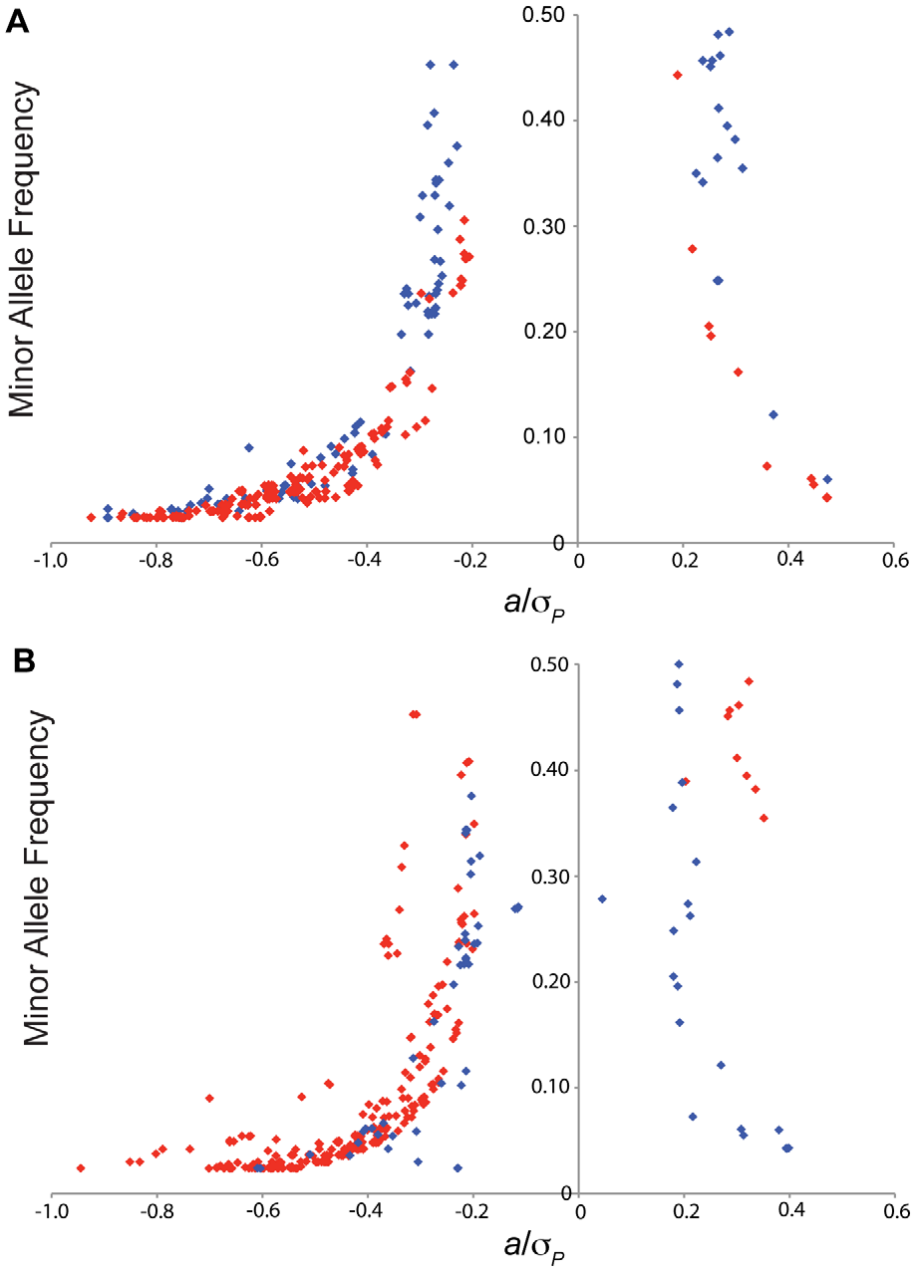
Minor allele frequency versus effect size. All main SNP effects and corresponding minor allele frequencies are shown. (A) Main SNP effects across sexes (females, males and sexes pooled) for each treatment. Survival on paraquat is depicted by red dots, survival on MSB by blue dots. (B) Main SNP across treatments (paraquat, MSB, and treatments pooled) effects for each sex. Females are depicted by red dots, males by blue dots.

The majority of SNPs associated with variation in susceptibility to oxidative stress were not common, and at the low end of the allele frequency spectrum amenable to association mapping (*i.e.*, we required the minor allele to be present in at least four DGRP lines). For the analyses of survival time on either paraquat and MSB pooled across sexes we found that the SNP effects (females, males and sexes pooled) were inversely related to minor allele frequencies, such that the less common SNPs had greater effects (Figure 4A; Table S5). The effect sizes were comparable for survival on both paraquat and MSB. For both treatments, negative effects (where flies homozygous for the minor allele live longer under oxidative stress than do flies homozygous for the major allele) greatly outnumbered positive effects. For the analyses of survival time of females and males pooled across treatments we also found that the SNP effects (survival time on paraquat, survival time on MSB, and treatments pooled) were inversely related to minor allele frequency (Figure 4B; Table S6). Female SNP effects were larger than those observed in males.

As expected from the quantitative genetic analyses indicating significant genetic variation in sexual dimorphism for survival on paraquat and MSB, we identified 87 (45) SNPs with significant SNP by sex interaction terms for paraquat (MSB) at *P*<10^−5^. Many of these SNPs exhibited antagonistic pleiotropy between the sexes. The minor allele was associated with increased survival in females and decreased survival in males for 51 of the SNPs with significantly different effects on survival on paraquat in males and females. The opposite pattern – the minor allele associated with decreased survival of females and increased survival of males – was observed for 38 of the SNPs with significantly different effects on survival on MSB in males and females. Only three SNPs were associated with decreased survival of females and increased survival of males on paraquat; and seven SNPs were associated with increased survival of females and decreased survival of males on MSB. Thus, all SNPs exhibiting SNP by sex interactions for survival on MSB had sexually antagonistic effects. The majority (62%) of SNPs with SNP by sex interactions for survival on paraquat had sexually antagonistic effects; the remainder had sex-biased or sex-specific effects. The larger number of SNPs affecting both sexes were associated with increased survival of females on paraquat and increased survival of males on MSB; this is consistent with the mean differences in male and female survival on paraquat and MSB.

Our quantitative genetic analysis also indicated significant genetic variation in the magnitude and or direction of the effects of the two treatments. Consistent with this observation, we identified 54 (21) SNPs exhibiting significant SNP by treatment interactions in females (males). In females, 34 of these SNPs (63%) had opposite effects in the two treatments, while in males 13 (62%) had opposite effects on survival under paraquat and MSB. The remaining SNPs with significant SNP by treatment interactions were treatment-specific or -biased.

**Figure 5:**
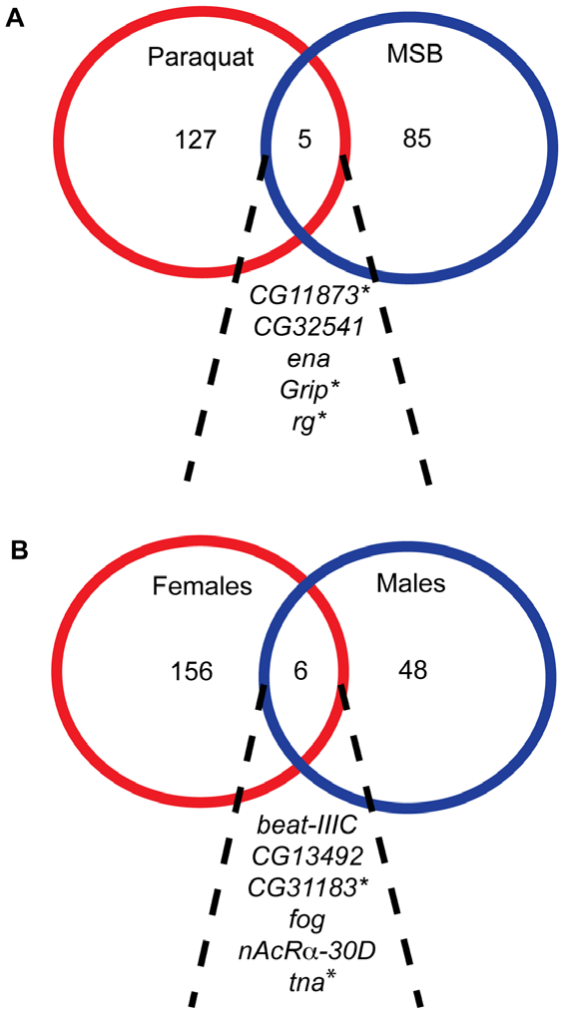
Overlap of genes with significant SNPs between treatments and sexes. The diagram shows genes with at least one significant SNP detected in genome-wide association analysis. (A) Survival time on paraquat and MSB (sexes pooled). (B) Females and males (treatments pooled). Genes with human homologs are indicated with an asterisk.

In our analyses pooled across sexes for each treatment separately (Table S5), we found no overlap at the level of SNPs associated with survival time on paraquat and MSB. However, different SNPs in five genes (*CG11873*, *CG32541*, *enabled* (*ena*), *Glutamate receptor binding protein* (*Grip*), and *rugose* (*rg*)) were associated with survival time on both paraquat and MSB (Figure 5A). Three of these genes (*CG11873*, *Grip* and *rg*) have human homologs (Table S7). In our analysis pooled across treatments for males and females separately we found four SNPs associated with variation in oxidative stress susceptibility in both sexes: *2L*_17233438 in the intron of *beat-IIIC*; *3R*_11695487, a missense mutation in *CG31183*; and *X*_22240772 and *X*_22240773, both in the intron of *folded gastrulation* (*fog*). Three additional genes, *CG13492*, *nicotine Acetylcholine Receptor α 30D* (*nAcRalpha-30D*) and *tonalli* (*tna*) contained different SNPs that were associated with variation in oxidative stress susceptibility in both sexes (Figure 5B). Two of these genes, *CG31183* and *tna*, have human homologs (Table S7).

### Gene-centered prediction models

Single marker association analysis leads to biased estimates of allelic effects when multiple SNPs affect the trait, and SNPs are correlated. We therefore computed gene-centered forward selection multiple regression models to estimate effect sizes when multiple SNPs are simultaneously evaluated in the model, and to estimate the fraction of the total variation accounted for by the SNPs. We computed the multiple regression models separately for paraquat and MSB, and for females and males as well as averaged across sexes within each treatment. We allowed a maximum of 12 SNPs in each model.

The proportion of phenotypic variation explained by the multiple regression models is given by the model *r* ^2^. The models explain a relatively large amount of phenotypic variation for survival under both paraquat (67–79%; Table 3) and MSB (56–66%; Table 4). We also estimated the fraction of the total genetic variance explained by the models as the fraction of the total variance among line means due to variance among haplotypes formed by the SNPs in the models. In all cases the genetic variance explained was high: 82% and 78% for females and males, respectively, for survival on paraquat; and 73% and 64% for females and males, respectively, for survival on MSB.

**Table 3:** Gene-centered predictive models of survival time on paraquat.

Analysis	Variable	SNP Location	Estimate	*F*	*P*-Value
Females*r^2^* = 0.7878	Intercept		12.989	1379.51	<0.0001
	*2R*_16842959	*hbn* (cds)	2.283	45.56	<0.0001
	*3L*_17705194	*Ccn* (in)	0.886	14.08	0.0003
	*2L*_10188836	*Sur* (cds)#	1.202	17.77	<0.0001
	*X*_9979448	*CG34104* (in)	−0.887	18.38	<0.0001
	*3L*_9610795	*LanB2* (in)	3.366	44.45	<0.0001
	*2L*_10499146	*Myo31DF* (in)	0.877	15.09	0.0002
	*3L*_15871676	*pHCl* (in)	2.515	28.74	<0.0001
	*3L*_17361008	*Cad74A* (u3)	1.126	12.61	0.0005
	*3R*_5549461	*hyd* (cds)#	1.687	14.61	0.0002
	*X_*16161887	*kat80* (in)	0.881	15.94	0.0001
	*3L*_292059	*RhoGEF3* (in)	1.582	9.22	0.0029
Males*r^2^* = 0.6748	Intercept		13.611	1409.36	<0.0001
	*2R*_11302152	*CG34356* (in)	−1.986	23.54	<0.0001
	*2L*_549825	*Ets21C* (in)	2.164	17.69	<0.0001
	*2L_*2659687	*Prosbeta4R2* (in)	−1.079	8.26	0.0047
	*2R_*11364415	*CG8180* (in)	2.460	28.45	<0.0001
	*X*_16161887	*kat80* (in)	0.843	14.02	0.0003
	*3R_*21907532	*Dys* (in)	−1.626	10.68	0.0014
	*X_*16287121	*mei-41* (cds)	−0.730	13.98	0.0003
	*3L_*17705194	*Ccn* (in)	0.749	10.46	0.0015
	*3R_*20053152	*CG13609* (cds)#	0.788	11.89	0.0008
	*3L_*602217	*CG13893* (in)	1.493	16.13	<0.0001
	*3L_*17173704	*Rbp6* (in)	−1.240	11.36	0.0010
	*2L_*925438	*CG4341* (in)	−0.780	6.81	0.0101

Markers are listed in the order in which they entered the model. Estimates of effects are for (Minor allele – Major allele). In: intronic; cds: coding sequence;

# : Missense; u3/5: 3′5′ UTR.

**Table 4:** Gene-centered predictive models of survival time on MSB.

Analysis	Variable	SNP Location	Estimate	*F*	*P*-Value
Females*r^2^* = 0.6606	Intercept		24.746	859.33	<0.0001
	*3L*_7755124	*Hn* (cds)	2.145	9.21	0.0029
	*X*_20477383	*RunxA* (in)	4.875	20.46	<0.0001
	*3L_*10866866	*Tna* (cds)#	5.035	17.17	<0.0001
	*2L*_8431541	*grk* (u3)	4.413	12.14	0.0007
	*3R_*11695487	*CG31183* (cds)#	−1.171	6.69	0.0108
	*2L_*4748714	*CG15630* (in)	1.183	6.64	0.0111
	*2R_*9461631	*CG42808* (cds)	−1.736	14.20	0.0002
	*2R_*11256183	*Pms2* (cds)	3.589	13.32	0.0004
	*X_*7115312	*CG9650* (in)	2.973	5.28	0.0232
	*2L_*6734157	*TTLL3B* (cds)	2.910	5.15	0.0248
	*3L_*3138481	*CG11537* (in)	−2.074	4.91	0.0284
Males*r^2^* = 0.5557	Intercept		28.741	1896.07	<0.0001
	*3R_*11695487	*CG31183*(cds)#	−1.887	19.82	<0.0001
	*X_*20477383	*RunxA* (in)	5.446	21.79	<0.0001
	*2L_*4897343	*CG3036*(in)	2.549	14.15	0.0002
	*2L_*6734157	*TTLL3B* (cds)	3.359	8.71	0.0037
	*3L_*3138481	*CG11537* (in)	−3.369	16.59	<0.0001
	*3L_*7755124	*Hn* (cds)	1.885	8.23	0.0047
	*3L_*1960704	*CG42863* (cds)	3.029	4.68	0.0323
	*2L_*25648943	*neo* (cds)	4.246	7.47	0.0071
	*X_*4539167	*HLH4C* (UTR)	1.985	4.93	0.0280

Markers are listed in the order in which they entered the model. Estimates of effects are for (Minor allele – Major allele). In: intronic; cds: coding sequence;

# : Missense; u3/5: 3′5′ UTR.

For paraquat, only two SNPs were included in both the female and male prediction models (*3L_*17705194, an intronic SNP in *Ccn*, and *X_*16161887 an intronic SNP in *katanin 80* (*kat80*)). For MSB, three SNPs were shared between the female and male prediction models (*X_*20477383, an intronic SNP in *RunxA*, *3R_*11695487, a missense mutation in *CG31183*, and *3L_*3138481 an intronic SNP in *CG11537*). These results show that alleles with large effects on oxidative stress resistance are generally not shared between the sexes. We found no overlap between the genes in the multiple regression models for survival on paraquat and on MSB, which highlights the low correlation in survival in response to the different agents.

### Validation of candidate genes

**Figure 6:**
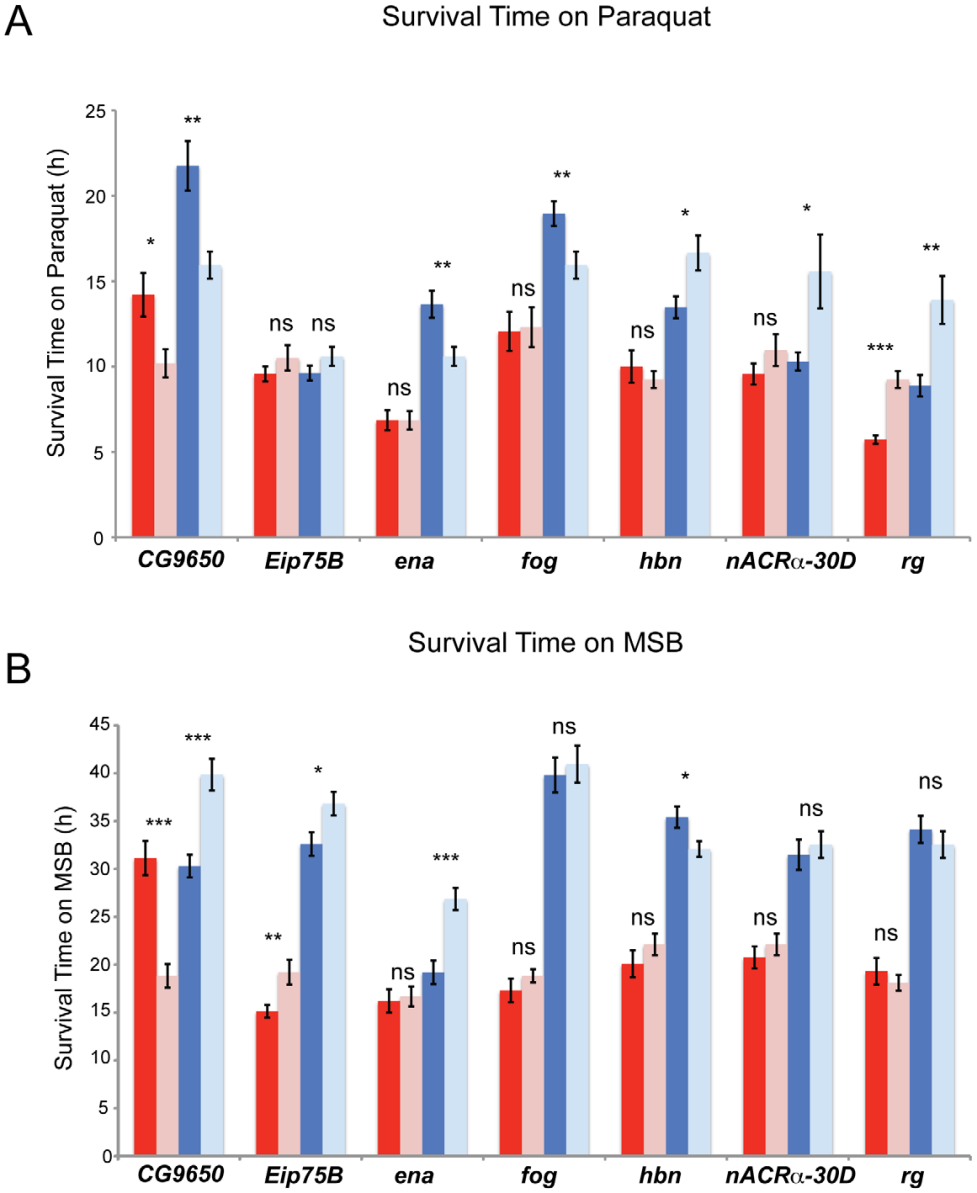
Validation of candidate oxidative stress susceptibility genes using mutants. Seven mutants *CG9650^BG01024^*, *Eip75B^BG02737^*, *ena^BG02189^*, *fog^BG01196^*, *hbn^MB04955^*, *nAcRα-30D^MB06675^*, and *rugose^MB01845^* were tested for each sex and treatment along with their corresponding control. The averages are color coded: red, female mutant; pink female control; blue, male mutant; light blue, male control. All mutants were homozygous. (A) Survival time on paraquat. (B) Survival time on MSB. *: 0.01≤*P*≤0.05; **: 0.001≤*P*≤0.01; ***: *P*<0.001; ns: *P*>0.05.

We selected seven candidate genes (*CG9650*, *Ecodysone-induced protein 75 B* (*Eip75B*), *ena*, *fog*, *homeobrain* (*hbn*), *nACRα-30D*, and *rg*) associated with oxidative stress susceptibility/resistance for mutant validation. These genes were chosen based on the significance level of their association with phenotypic variation and on the availability of co-isogenic *P*-element or *Minos*-element mutant alleles. All seven genes and corresponding controls were tested for both sexes with both treatments. All seven genes showed a significant difference in survival time on an oxidizing agent between mutant and control for at least one sex (Figure 6). In total, 14 tests were significant (Table S8), greater than the number expected by chance (1.4, Fisher's exact test, *P* = 0.0001).

### A cellular network controlling oxidative stress susceptibility

**Figure 7:**
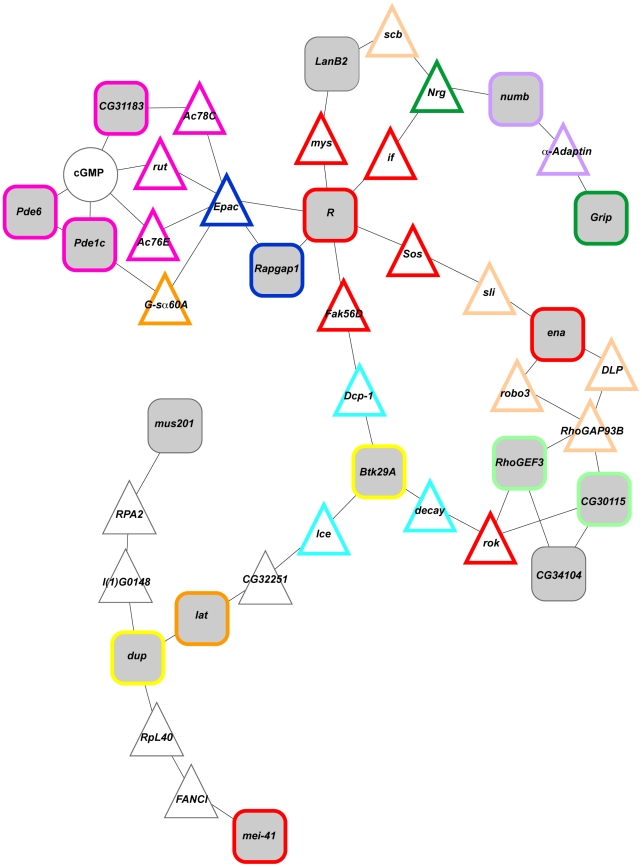
A cellular network among candidate genes. This diagram depicts an enriched network (*P* = 0.005) among candidate genes with at least one significant SNP detected in GWA analysis. Candidate genes are indicated by grey filled squares, missing genes (*i.e*., genes without significant associations) by white filled triangles and metabolites by white filled circles. Gene ontology categories are represented by boarders other than black (magenta, purine metabolism; peach, axon guidance; aqua, apoptosis; yellow, DNA endoreduplication; purple, asymmetric cell division; navy blue, regulation of small GTPase mediated signal transduction; dark green, synapse organization; orange, learning or memory; light green, regulation of Rho protein signal transduction).

In order to assess to what extent genes associated with oxidative stress susceptibility encode products that interact in common cellular pathways, we performed a bioinformatics analysis using the RSpider algorithm [31]. Rspider incorporates knowledge from both the Reactome signaling network and KEGG metabolic network to determine if interactions are overrepresented compared to that expected by chance. Using a model that allowed for two missing genes (*i.e.*, genes that connect our candidate genes in Reactome signaling or KEGG metabolic networks) and/or compounds between our candidate genes, we identified a network significantly enriched (*P* = 0.005) for 42 genes that includes 17 candidate genes from the genome wide association analysis (Figure 7). Thirty-one of the 42 genes in this network have human homologs. These genes were associated with gene ontology categories of purine metabolism, axon guidance, apoptosis, DNA endoreduplication, asymmetric cell division, regulation of small GTPase mediated signal transduction, synapse organization, learning or memory and regulation of Rho protein signal transduction. These gene ontology categories reflect oxidative stress susceptibility of DNA metabolism and neuronal function.

## Discussion

Our study represents the largest effort to date in utilizing genome wide natural genetic variation in a model system to uncover the genetic basis of susceptibility/resistance to oxidative stress. By conducting genome wide association analyses using the DGRP we were able to gain insight into the genetic architecture of oxidative stress susceptibility and identify novel genes associated with variation in this complex trait as well as a network that highlights the impact of oxidative stress susceptibility on DNA metabolism and neuronal development.

### GWA for susceptibility to oxidative stress

Our single SNP association analyses identified many novel candidate genes and genetic variants associated with acute oxidative stress susceptibility/resistance in *D. melanogaster*. Few of the genes identified using naturally occurring variation have been previously implicated in oxidative stress response, and many of the genes known from other studies to affect oxidative stress susceptibility were not identified in this study. For example, neither *Sod* nor *Cat*, which are major players in the removal of ROS [15], [16], [17], [18] harbor SNPs associated with variation in oxidative stress susceptibility, possibly because these loci are under strong purifying selection or because SNPs in these loci were too rare to be included in the association analysis. Thus, association mapping using variants that have survived the sieve of natural selection complements biochemical and mutant analyses to understand the genetic architecture of quantitative traits.

In contrast to results from human association studies [32], multiple regression models incorporating up to 12 genic SNPs explain a large proportion of the phenotypic and genetic variance in oxidative stress resistance. Further, we find an inverse relationship between minor allele frequency and effect size, such that less common alleles have the largest effects. If low frequency alleles similarly have the largest effects on complex diseases and traits in human populations, they will be poorly tagged by LD with the common SNPs used in association studies. This could at least partially account for the ‘missing heritability’ [32] in human GWA analyses.

We note that much variation for fitness traits in natural populations is thought to be attributable to mutation-selection balance [29], and that oxidative stress susceptibility is a fitness related trait interconnected with other fitness traits such as fecundity and lifespan [33]. Under the mutation-selection balance model we expect an inverse relationship between the magnitude of the effect on fitness and allele frequency, as observed. The interconnectedness between oxidative stress susceptibility and fitness related traits could also explain why the majority of our significantly associated SNPs have negative effects (homozygotes for the minor allele live longer under oxidative stress than do homozygotes for the major allele). Since we postulate that increased resistance to oxidative stress should be positively correlated with reproductive fitness, we hypothesize that these alleles have not reached higher frequencies in the population because they have negative correlations with other fitness related traits.

### Oxidative stress susceptibility is sexually dimorphic

Oxidative stress susceptibility within the DGRP is sexually dimorphic (Table 1; Figure 1), consistent with previous work in *D. melanogaster* showing that rescue of SOD-deficient flies by exogenous antioxidants was sex-specific [34]. Similarly, differences in oxidative stress susceptibility between males and females have been documented in humans [35], [36], [37] and it has been hypothesized that this could account for differences in susceptibility to cardiovascular disease in men and women [38]. The nature of the sexual dimorphism we observed was dependent on the oxidative stress inducing agent, with females surviving longer than males on paraquat, and males surviving longer than females on MSB (Figure 1).

In addition to sexual dimorphism in susceptibility to oxidative stress averaged over all DGRP lines, there is also genetic variation in the magnitude and direction of the difference in oxidative stress susceptibility between males and females. Many SNPs with significant differences between males and females had sexually antagonistic effects. This is interesting from an evolutionary perspective, since antagonistic pleiotropic effects in males and females can lead to maintenance of variation for fitness [29]. Indeed, 40% (26%) of the SNPs with opposite effects in males and females for survival on MSB (paraquat) had minor allele frequencies greater than 0.15, consistent with this hypothesis. These sexually dimorphic genes may drive the greater susceptibility of males to paraquat and the greater susceptibility of females to MSB.

### Oxidative stress susceptibility is dependent on the method of oxidative stress induction

Oxidative stress candidate genes identified by our study were generally specific to either paraquat or MSB treatment (Figure 5A). Similarly, previous studies in *Drosophila* [24] and yeast [39], [40] found distinct transcriptional responses to different oxidizing treatments. These observations are consistent with the different modes of actions through which these compounds do damage to the cell. Experimental evidence suggests that the toxicity of paraquat is primarily due to redox-cycling, as opposed to menadione sodium bisulfite (a water soluble derivative of menadione), whose toxicity has been found to be predominantly due to mechanisms other than superoxide production, such as electrophilic attack [41]. Other factors that could account for the lack of overlap between the two treatments include feeding propensity and metabolism. However, we also identified 34 (13) SNPs with significant SNP by treatment effects that had effects of opposite sign in females (males). These SNPs exhibit antagonistic pleiotropy for genotype by environment interaction, another mechanism for maintaining genetic variance for fitness in a natural population. Consistent with this hypothesis, 63% (62%) of the SNPs with opposite effects on survival on paraquat and MSB had minor allele frequencies greater than 0.15 in females (males).

### Functional tests

We focused our efforts to validate the effects of candidate genes identified by GWA using mutations in genes that were in common between males and females and both treatments, as well as in genes with low *P*-values. We tested two genes (*fog*, *nACRα-30D*) that were significantly associated with oxidative stress susceptibility in both sexes; two genes (*ena*, *rg*) that were significantly associated with oxidative stress susceptibility in both treatments; and three genes (*CG9650*, *Eip75B*, *hbn*) with low *P*-values. Mutations in all seven genes were significant in at least one sex or treatment (Figure 6). *fog* is involved in the torso signaling pathway and regulates cell shape [42]. *Eip75B* is a nuclear receptor that is involved in the signaling action of nitric oxide [43], a free radical that has neuroprotective properties at moderate to low concentrations and neurotoxic properties at high concentrations [44]. *nAcRα-30D* has neurotransmitter receptor activity [45] and has been implicated in insecticide resistance [46]. *ena* has been implicated in many biological processes, including dendritic morphogenesis [47], [48]. *rg* encodes an A-kinase anchoring protein [49] involved in signal integration in neurons and memory processing [50]. The human homolog of *rg*, *NBEA*, has been implicated in myeloma [51] and autism [52], [53]. *CG9650* is a zinc-finger-containing putative transcription factor that is a modifier of Notch signaling [54] and is involved in axon guidance [55]. *hbn* is a homeobox transcription factor that is expressed in the brain [56]. Since *Eip75B*, *nAcRα-30D*, *ena*, *rg*, *CG9650*, and *hbn* have neurological function or are expressed in the brain, they are of particular interest given the relationship between oxidative stress susceptibility and neurodegenerative disease in humans [57]. Thus, genes that are associated with oxidative stress induced by multiple agents may represent common oxidative stress targets associated with function of the nervous system.

### Candidate oxidative stress target genes converge on a cellular network

We identified a cellular network comprising 42 genes, including 17 of our candidate genes, centered on DNA metabolism and neural development (Figure 7). Twelve of the candidate genes have human homologs, ten of which have been implicated in human diseases. Two genes, *CG31183* and *Grip*, are associated with susceptibility to oxidative stress induced by both paraquat and MSB. *CG31183* is predicted to be involved in protein phosphorylation and intracellular signal transduction [58] and its human homolog, *NPR1*, has been implicated in hypertension and cardiovascular disease [59], [60]. *Grip* encodes a glutamate receptor binding protein involved in synapse organization [61] and its human homolog, *GRIP2*, has been implicated in Alzheimer's disease [62]. *LanB2* and *Roughened* (*R*) also have human homologs implicated in Alzheimer's disease [63], [64]. Another gene with neuronal function, *lat*, has a human homolog that has been associated with schizophrenia [65]. *Rapgap1* and *numb* have human homologs with tumor suppressor activity [66], [67], [68] and two DNA repair genes, *mei-41* and *mus201* [69], [70], have human homologs associated with Seckel syndrome [71] and xeroderma pigmentosum VII [72]. Lastly, the human homolog of *Btk29A* has been implicated in gout [73].

### Conclusions and future directions

We have shown that the genetic architecture of oxidative stress susceptibility in *Drosophila* is complex, sexually dimorphic and dependent on the oxidative stress inducing agent. Candidate genes associated with oxidative stress susceptibility/resistance fall largely in gene ontology categories associated with DNA metabolism and nervous system development and function. While we were successful in identifying many SNPs associated with oxidative stress resistance, some of the completely recessive alleles detected in our study presumably do not contribute to variation in outbred populations. Future work using progeny from crosses and outbred populations will be necessary in order to detect dominance effects that contribute to oxidative stress susceptibility, resulting in a more complete understanding of the genetic architecture underlying this trait. This study and future work regarding oxidative stress susceptibility/resistance in *D. melanogaster* allow the identification of evolutionarily conserved target genes and gene networks that can serve as a blueprint for future translational studies on oxidative stress in people.

## Materials and Methods

### *Drosophila* stocks

We used 167 inbred lines of the *Drosophila melanogaster* Genetic Reference Panel (DGRP) [28]. This panel was derived from the Raleigh, USA population by 20 generations of full-sib inbreeding from isofemale lines derived from single inseminated wild-caught females. Flies were reared at a controlled density on cornmeal-molasses-agar medium at 25°C, 60–75% relative humidity and a 12-h light-dark cycle.

### Survival time on oxidative stress inducing agents

We measured 24 three-six day-old individual flies per sex per DGRP line per oxidative stress inducing agent for 88 hours using the *Drosophila* Activity Monitoring System (Trikinetics). Time of death was defined as the last activity count followed by six hours of inactivity. Survival times were expressed as the deviation from a contemporaneous *w* ^1118^; *Canton-S* isogenic control line mean. Two oxidative stress inducing agents were administered through diet, paraquat (1,1′-dimethyl-4,4′-bipyridinium dichloride) and menadione sodium bisulfite (MSB). To assay survival time on paraquat, individual flies were exposed to filter paper soaked with 50 µL of a 1% sucrose solution containing 20 mM paraquat. Previous studies showed that a 20 mM paraquat 1% sucrose solution is sufficient to induce oxidative stress related death in *Drosophila* within 88 hours [25], [74]. To assay survival time on MSB, individual flies were exposed to cornmeal-molasses-agar medium containing 75 mM MSB. Pilot experiments using a subset of the DGRP lines established that cornmeal-molasses-agar medium containing 75 mM MSB had a similar severity to that of the paraquat treatment, that is, this treatment also resulted in oxidative stress-induced death within 88 hours. For both treatments flies were placed in individual tubes with cornmeal-molasses-agar medium for 24 h prior to the assay, food deprived on 1.5% agar for two hours, and subsequently exposed to 20 mM paraquat or 75 mM MSB in individual tubes. All treatments were initiated between 9:00 am and 12:00 pm at 25°C and 70% humidity.

### Quantitative genetic analyses

We assessed the effect of *Wolbachia* infection status, previously measured on the DGRP lines [28], on survival time on paraquat and MSB using factorial, mixed model ANOVAs. The model used was *Y = μ+S+I+S×I+L(I)+S×L(I)+ε*, where *I* denotes the fixed effect of infection status, *S* is the fixed effect of sex, *L* is the random effect of the DGRP line, and *ε* is the error variance. We also performed reduced analyses for each sex separately. In cases where the effect of *Wolbachia* was significant, data were corrected, separately by sex.

We partitioned phenotypic variance using the *Wolbachia*-corrected data using ANOVAs of two forms. For Model 1, both treatments were analyzed separately using an ANOVA of the form *Y* = *μ+S+L+S×L+ε*, where terms are defined as above. Reduced ANOVAs were also performed for each sex separately. For Model 2, treatments were pooled and sexes were analyzed separately using an ANOVA of the form *Y* = *μ+T+L+T×L+ε*, where *T* is the fixed effect of treatment. For Model 1, we estimated broad-sense heritabilities (*H^2^*) as *H^2^* = (*σ^2^_L_*+*σ^2^_SL_*)/(*σ^2^_L_*+*σ^2^_SL_*+*σ^2^_E_*), where *σ^2^_L_*, *σ^2^_SL_*, and *σ^2^_E_* are the among-line, sex by line and within-line variance components, respectively. Similarly, broad-sense heritabilities were estimated for Model 2 as *H^2^* = (*σ^2^_L_*+*σ^2^_TL_*)/(*σ^2^_L_*+*σ^2^_TL_*+*σ^2^_E_*), where *σ^2^_TL_* is the line by treatment interaction. Coefficients of genetic (*CV_G_* = 100*σ_G_*/mean) and environmental (*CV_E_* = 100*σ_E_*/mean) variance were also computed. For the analyses of each sex separately, *H* ^2^ = *σ_L_* ^2^/(*σ_L_* ^2^+*σ_W_* ^2^), where *σ_L_* ^2^ is the among line variance component for males or females. We estimated cross-trait (cross-sex) genetic correlations as *r_G_ = cov_ij_/σ_i_σ_j_*, where *cov_ij_* is the covariance of line means between traits *i* and *j* (males and females), and *σ_i_* and *σ_j_* are the square roots of the among line variance components for the two traits (males and females).

### Genome-wide association analyses

We tested survival times on paraquat and MSB for association with 2,481,491 SNPs previously identified by whole-genome sequencing of the DGRP lines [28]. All analyses were performed on line means, adjusted for the effect of *Wolbachia* infection status. All segregating sites within lines were treated as missing data. SNPs were filtered based on several criteria: (1) the minor allele had to be represented in at least four DGRP lines; (2) SNPs were excluded if coverage from whole-genome sequencing was less than 2 or greater than 30 [28]; (3) SNPs with more than two segregating alleles within the 167 DGRP lines were excluded from analysis and; (4) SNPs had to be genotyped in at least sixty of the 167 DGRP lines.

Each SNP was tested for association with survival time on paraquat and MSB using an ANOVA of the form *Y = μ+M+S+M×S+L(M)+ε*, where *M* is the effect of the SNP, and *S* and *L* are as defined above. Reduced analyses of the form *Y = μ+M+ε* were also performed for males and females separately. Analyses where paraquat and MSB treatments (*T*) were pooled separately for each sex using the ANOVA model *Y = μ+M+T+M×T+L(T)+ε*, were also performed. The main effect (*a*) of each SNP was estimated as one-half the difference in trait mean between marker classes (polarized by allele frequency, such that the effect is the difference between the major and minor alleles) [29]. For analyses pooled across sexes, the interaction effect between SNP and sex was calculated as the difference between the female and male effects. Similarly, for analyses pooled across treatments, the interaction between SNP and treatment was calculated as the difference between the paraquat and MSB effect.

### Gene-centric forward selection models

Gene-centered forward selection was used to generate multiple regression models in order to identify SNPs that were predictive for survival on paraquat and MSB. Only significant SNPs (*P*<10^−5^) that were within a gene were incorporated into the model. Only one SNP was included in the model for pairs of SNPs in high LD. The most significant gene-centered SNP was fitted in the model first, and markers were sequentially added until the maximum *r^2^* for variance explained was reached, up to a maximum of 12 markers. Models were fitted for each treatment and performed separately using line mean data from females, males and the average of the two sexes. Once a final model was selected, an ANOVA of the form *Y* = *μ*+*H*+*L(H)*+*ε* was performed, where *H* denotes haplotype and *L* line. The fraction of the total genetic variance accounted for by the model was estimated as *σ_H_* ^2^/(*σ_H_* ^2^+*σ_L_* ^2^), where *σ_H_* ^2^ is the among-haplotype variance component and *σ_L_* ^2^ is the among-line variance component.

### Mutant validation

For seven of the candidate oxidative stress response genes identified in the GWA study, we tested *P*-element and *Minos*-element mutations and co-isogenic control lines for effects on oxidative stress resistance. *P*-element and *Minos*-element insert lines and their co-isogenic controls were obtained from the Berkeley *Drosophila* Gene Disruption Project [75] and the *Drosophila* Gene Disruption Project [76]. Specific alleles tested were *CG9650^BG01024^*, *Eip75B^BG02737^*, *ena^BG02189^*, *fog^BG01196^*, *hbn^MB04955^*, *nAcRα-30D^MB06675^*, and *rugose^MB01845^*. For each mutant and corresponding control, between 29 and 32 flies were measured for each sex and treatment, using the assays described above.

### Bioinformatics

Statistical analyses were performed using SAS software (SAS, Cary, NC, USA). Functional annotations of genes are based on Flybase [77].

## Supporting Information

Table S1 *Wolbachia* infection status and mean phenotypic values of paraquat and MSB resistance.
(DOC)

Table S2 Analyses of variance of survival time on paraquat and MSB.
(DOC)

Table S3 Analyses of variance of females and males pooled across treatments.
(DOC)

Table S4 Quantitative genetic analysis of females and males pooled across treatments.
(DOC)

Table S5 GWA analysis results for survival time on paraquat and MSB (sexes pooled).
(XLS)

Table S6 GWA analysis results for females and males (treatments pooled).
(XLS)

Table S7 Candidate genes with human homologs and their role in complex disease.
(XLS)

Table S8 Validation mutant test *P*-values.
(DOC)
