# Supplementary material for: Genome-Wide Association Analysis of Oxidative Stress Resistance in Drosophila melanogaster
Source: PLoS One. 2012 Apr 4;7(4):e34745. doi: 10.1371/journal.pone.0034745 (PMC3319608; doi:10.1371/journal.pone.0034745)
Supplement: Table S1 — Wolbachia infection status and mean phenotypic values of paraquat and MSB resistance. (DOC) [file pone.0034745.s001.doc]

**Table S1.** *Wolbachia* infection status and mean phenotypic values of paraquat and MSB resistance.

*Wolbachia* infection status (WI) and mean phenotypic values for paraquat resistance (PR), and MSB resistance (MR), separately for females (F) and males (M) in the DGRP lines. *, Means adjusted for the effect of *Wolbachia* infection.

| **DGRP Line** | **WI** | **PR F*** | **PR M*** | **MR F** | **MR M*** |
| --- | --- | --- | --- | --- | --- |
| RAL_21 | Y | 7.450 | 7.681 | 19.691 | 26.060 |
| RAL_26 | N | 10.706 | 10.144 | 21.908 | 22.276 |
| RAL_28 | N | 11.710 | 12.970 | 17.380 | 21.093 |
| RAL_38 | N | 11.234 | 8.674 | 21.819 | 25.773 |
| RAL_40 | Y | 22.316 | 16.007 | 35.518 | 37.520 |
| RAL_41 | N | 15.932 | 16.613 | 40.428 | 34.511 |
| RAL_42 | N | 8.229 | 7.222 | 20.695 | 22.923 |
| RAL_45 | N | 16.002 | 20.014 | 21.326 | 22.500 |
| RAL_49 | Y | 10.692 | 13.305 | 32.255 | 38.163 |
| RAL_57 | N | 12.345 | 10.388 | 17.877 | 15.626 |
| RAL_59 | N | 12.049 | 13.742 | 15.416 | 17.892 |
| RAL_69 | Y | 9.016 | 8.947 | 13.986 | 34.464 |
| RAL_73 | Y | 7.742 | 5.799 | 20.018 | 31.977 |
| RAL_75 | Y | 12.790 | 12.470 | 17.112 | 31.086 |
| RAL_83 | N | 12.047 | 16.999 | 34.255 | 32.533 |
| RAL_85 | N | 7.304 | 7.826 | 17.325 | 21.249 |
| RAL_88 | N | 16.023 | 16.161 | 24.192 | 28.950 |
| RAL_91 | N | 16.322 | 15.761 | 37.291 | 37.496 |
| RAL_93 | N | 20.792 | 18.608 | 31.970 | 25.946 |
| RAL_101 | N | 19.666 | 16.021 | 19.658 | 21.075 |
| RAL_105 | N | 25.041 | 14.309 | 17.487 | 14.801 |
| RAL_109 | N | 20.130 | 16.803 | 18.701 | 20.689 |
| RAL_129 | N | 17.280 | 14.233 | 22.513 | 21.491 |
| RAL_136 | Y | 19.313 | 19.428 | 32.566 | 43.389 |
| RAL_138 | N | 12.743 | 13.732 | 28.756 | 31.041 |
| RAL_142 | Y | 16.006 | 12.383 | 27.999 | 36.354 |
| RAL_149 | Y | 14.111 | 16.446 | 20.967 | 30.268 |
| RAL_153 | Y | 10.263 | 8.093 | 40.283 | 41.002 |
| RAL_158 | N | 16.664 | 13.203 | 46.267 | 36.076 |
| RAL_161 | N | 11.596 | 8.195 | 18.895 | 14.320 |
| RAL_176 | Y | 16.262 | 8.891 | 24.765 | 18.877 |
| RAL_177 | N | 21.392 | 14.013 | 24.804 | 33.345 |
| RAL_181 | Y | 9.697 | 11.904 | 22.060 | 25.509 |
| RAL_195 | N | 21.641 | 21.698 | 17.949 | 17.960 |
| RAL_208 | N | 18.708 | 21.677 | 22.050 | 26.040 |
| RAL_217 | N | 17.597 | 13.955 | 18.176 | 20.822 |
| RAL_227 | Y | 14.057 | 14.177 | 24.854 | 25.859 |
| RAL_228 | N | 25.193 | 18.833 | 28.765 | 25.005 |
| RAL_229 | N | 11.262 | 10.050 | 17.406 | 21.216 |
| RAL_233 | N | 14.059 | 12.144 | 26.592 | 26.412 |
| RAL_235 | N | 14.720 | 9.763 | 33.483 | 24.362 |
| RAL_237 | Y | 23.325 | 18.204 | 41.171 | 44.544 |
| RAL_239 | N | 17.562 | 13.290 | 35.782 | 32.105 |
| RAL_256 | Y | 15.459 | 17.324 | 45.853 | 36.408 |
| RAL_280 | Y | 18.656 | 15.975 | 31.890 | 36.609 |
| RAL_287 | Y | 13.342 | 9.776 | 19.898 | 29.832 |
| RAL_301 | N | 13.549 | 11.374 | 25.579 | 27.730 |
| RAL_303 | N | 11.688 | 9.003 | 21.944 | 27.464 |
| RAL_304 | Y | 17.592 | 13.215 | 30.949 | 34.855 |
| RAL_306 | Y | 15.288 | 12.760 | 35.613 | 39.987 |
| RAL_307 | N | 14.370 | 12.396 | 22.805 | 17.951 |
| RAL_309 | N | 10.791 | 9.976 | 33.642 | 36.202 |
| RAL_310 | Y | 15.633 | 13.765 | 23.268 | 28.426 |
| RAL_313 | N | 15.224 | 12.957 | 38.706 | 31.758 |
| RAL_315 | N | 15.742 | 15.157 | 25.058 | 26.976 |
| RAL_317 | Y | 15.369 | 12.470 | 24.659 | 21.676 |
| RAL_318 | Y | 18.248 | 14.820 | 29.372 | 34.595 |
| RAL_320 | Y | 18.710 | 12.128 | 29.681 | 31.188 |
| RAL_321 | Y | 9.418 | 11.145 | 16.110 | 27.319 |
| RAL_324 | N | 11.699 | 13.820 | 17.619 | 18.894 |
| RAL_325 | N | 13.506 | 12.287 | 31.697 | 36.087 |
| RAL_332 | N | 13.108 | 16.220 | 18.922 | 25.744 |
| RAL_335 | Y | 7.706 | 4.832 | 22.049 | 17.549 |
| RAL_338 | Y | 17.853 | 11.445 | 21.540 | 26.132 |
| RAL_350 | N | 14.969 | 12.586 | 27.841 | 29.495 |
| RAL_352 | Y | 13.422 | 9.277 | 19.805 | 29.219 |
| RAL_356 | Y | 13.784 | 13.944 | 30.052 | 29.178 |
| RAL_357 | N | 16.251 | 13.638 | 21.904 | 22.081 |
| RAL_358 | N | 20.042 | 16.330 | 21.872 | 24.035 |
| RAL_359 | N | 15.138 | 17.105 | 20.102 | 35.535 |
| RAL_360 | Y | 11.332 | 14.711 | 34.331 | 34.267 |
| RAL_362 | Y | 26.056 | 21.990 | 33.541 | 33.485 |
| RAL_365 | Y | 13.254 | 12.334 | 29.439 | 28.430 |
| RAL_367 | N | 13.079 | 8.588 | 43.775 | 36.574 |
| RAL_370 | Y | 14.134 | 12.241 | 18.873 | 24.082 |
| RAL_371 | N | 14.131 | 11.218 | 21.273 | 23.544 |
| RAL_373 | N | 20.594 | 19.360 | 37.452 | 39.552 |
| RAL_374 | Y | 11.842 | 16.041 | 14.193 | 23.365 |
| RAL_375 | N | 17.496 | 10.003 | 40.437 | 37.895 |
| RAL_377 | N | 12.145 | 13.556 | 25.040 | 24.724 |
| RAL_379 | N | 17.686 | 11.854 | 27.650 | 19.778 |
| RAL_380 | Y | 13.895 | 14.644 | 36.326 | 35.577 |
| RAL_381 | N | 12.552 | 11.750 | 23.171 | 25.209 |
| RAL_383 | Y | 11.153 | 16.013 | 21.451 | 33.374 |
| RAL_386 | N | 11.005 | 8.138 | 17.582 | 20.101 |
| RAL_391 | N | 18.053 | 11.605 | 29.064 | 26.452 |
| RAL_392 | N | 12.951 | 10.724 | 39.399 | 30.064 |
| RAL_399 | N | 13.541 | 12.988 | 24.161 | 20.783 |
| RAL_405 | Y | 22.589 | 19.409 | 34.797 | 44.031 |
| RAL_406 | N | 15.876 | 19.645 | 31.033 | 30.768 |
| RAL_409 | Y | 13.234 | 10.161 | 21.126 | 31.433 |
| RAL_426 | N | 11.495 | 12.101 | 17.255 | 28.289 |
| RAL_427 | N | 14.646 | 11.932 | 17.851 | 21.511 |
| RAL_437 | N | 13.463 | 11.512 | 15.547 | 17.350 |
| RAL_439 | N | 13.224 | 15.461 | 30.380 | 34.864 |
| RAL_440 | Y | 21.105 | 13.967 | 29.953 | 33.020 |
| RAL_441 | Y | 16.000 | 17.391 | 21.202 | 41.324 |
| RAL_443 | N | 16.418 | 14.298 | 21.524 | 25.474 |
| RAL_461 | Y | 9.140 | 12.445 | 28.720 | 36.665 |
| RAL_486 | Y | 28.811 | 16.253 | 18.638 | 16.307 |
| RAL_491 | N | 25.865 | 21.338 | 36.728 | 33.272 |
| RAL_492 | N | 22.280 | 17.423 | 21.206 | 26.399 |
| RAL_502 | N | 13.527 | 13.169 | 24.535 | 24.903 |
| RAL_508 | N | 12.575 | 16.334 | 31.686 | 36.096 |
| RAL_509 | N | 14.096 | 13.793 | 21.618 | 26.985 |
| RAL_513 | Y | 12.672 | 12.120 | 24.894 | 27.687 |
| RAL_517 | N | 22.074 | 13.503 | 20.698 | 19.405 |
| RAL_531 | Y | 11.769 | 13.372 | 19.769 | 25.697 |
| RAL_535 | Y | 9.995 | 11.120 | 20.669 | 33.492 |
| RAL_555 | Y | 16.739 | 7.493 | 24.803 | 22.949 |
| RAL_563 | N | 15.623 | 14.336 | 25.958 | 43.089 |
| RAL_589 | Y | 27.183 | 20.595 | 25.022 | 27.001 |
| RAL_595 | Y | 10.526 | 11.126 | 24.039 | 30.701 |
| RAL_639 | Y | 13.518 | 14.064 | 24.129 | 25.977 |
| RAL_642 | N | 23.876 | 17.411 | 38.344 | 39.197 |
| RAL_646 | Y | 16.182 | 16.637 | 24.687 | 22.196 |
| RAL_703 | N | 11.004 | 15.658 | 9.759 | 21.733 |
| RAL_705 | Y | 29.929 | 21.584 | 45.698 | 40.928 |
| RAL_707 | Y | 8.764 | 7.980 | 27.836 | 24.137 |
| RAL_712 | Y | 17.043 | 14.536 | 19.589 | 23.145 |
| RAL_714 | N | 18.971 | 11.688 | 26.0492 | 21.204 |
| RAL_716 | Y | 18.285 | 19.745 | 22.155 | 32.808 |
| RAL_721 | Y | 15.579 | 12.689 | 31.110 | 41.472 |
| RAL_727 | Y | 17.312 | 16.091 | 16.866 | 28.765 |
| RAL_730 | Y | 31.909 | 22.676 | 47.901 | 40.115 |
| RAL_732 | N | 10.972 | 2.917 | 24.160 | 21.901 |
| RAL_737 | Y | 15.376 | 17.508 | 20.657 | 39.808 |
| RAL_738 | Y | 6.388 | 12.322 | 24.009 | 34.430 |
| RAL_757 | N | 10.226 | 10.614 | 17.825 | 24.284 |
| RAL_761 | Y | 12.531 | 14.178 | 11.962 | 30.425 |
| RAL_765 | N | 10.318 | 9.103 | 14.325 | 15.646 |
| RAL_774 | N | 11.187 | 13.850 | 18.371 | 18.979 |
| RAL_776 | Y | 19.609 | 14.782 | 25.063 | 31.624 |
| RAL_783 | Y | 12.259 | 18.169 | 27.752 | 43.677 |
| RAL_786 | Y | 13.507 | 11.938 | 22.539 | 25.576 |
| RAL_787 | Y | 15.698 | 14.439 | 20.674 | 35.490 |
| RAL_790 | Y | 17.552 | 17.506 | 37.965 | 30.316 |
| RAL_796 | Y | 11.283 | 11.983 | 14.649 | 39.266 |
| RAL_799 | N | 15.184 | 10.364 | 16.627 | 18.667 |
| RAL_801 | Y | 14.006 | 12.508 | 22.523 | 24.862 |
| RAL_802 | Y | 15.267 | 16.230 | 18.127 | 32.112 |
| RAL_804 | Y | 10.167 | 14.832 | 14.211 | 30.736 |
| RAL_805 | Y | 20.434 | 16.205 | 29.169 | 34.432 |
| RAL_808 | N | 17.037 | 16.909 | 31.132 | 27.063 |
| RAL_810 | N | 13.426 | 19.193 | 24.659 | 36.724 |
| RAL_812 | N | 16.779 | 16.278 | 27.674 | 29.079 |
| RAL_818 | Y | 9.114 | 10.214 | 18.347 | 27.946 |
| RAL_820 | Y | 28.174 | 13.860 | 42.904 | 34.709 |
| RAL_822 | Y | 16.837 | 12.869 | 18.565 | 29.936 |
| RAL_832 | Y | 18.698 | 16.657 | 23.113 | 23.913 |
| RAL_837 | Y | 16.040 | 16.542 | 19.616 | 32.402 |
| RAL_852 | Y | 9.489 | 4.030 | 34.787 | 31.709 |
| RAL_855 | Y | 15.940 | 18.787 | 47.880 | 47.053 |
| RAL_857 | N | 27.166 | 25.918 | 36.566 | 39.329 |
| RAL_859 | Y | 16.011 | 16.075 | 38.018 | 48.337 |
| RAL_861 | Y | 15.543 | 14.250 | 31.120 | 34.258 |
| RAL_879 | Y | 18.254 | 19.126 | 33.771 | 35.119 |
| RAL_882 | Y | 17.148 | 15.849 | 24.404 | 33.662 |
| RAL_884 | Y | 11.683 | 17.875 | 12.282 | 31.713 |
| RAL_887 | Y | 13.682 | 9.740 | 12.804 | 20.336 |
| RAL_890 | Y | 13.265 | 12.112 | 22.858 | 29.825 |
| RAL_892 | Y | 20.557 | 15.471 | 16.084 | 31.795 |
| RAL_894 | N | 21.713 | 20.744 | 23.538 | 22.457 |
| RAL_897 | Y | 15.767 | 12.807 | 15.101 | 26.495 |
| RAL_907 | N | 13.464 | 15.813 | 15.856 | 26.158 |
| RAL_908 | N | 16.575 | 18.858 | 20.597 | 19.761 |
| RAL_911 | N | 13.065 | 13.242 | 17.076 | 27.946 |
