# Supplementary material for: Genome-Wide Association Analysis of Oxidative Stress Resistance in Drosophila melanogaster
Source: PLoS One. 2012 Apr 4;7(4):e34745. doi: 10.1371/journal.pone.0034745 (PMC3319608; doi:10.1371/journal.pone.0034745)
Supplement: Table S2 — Analyses of variance of survival time on paraquat and MSB. (DOC) [file pone.0034745.s002.doc]

**Table S2. Analyses of variance of survival time on paraquat and MSB.**

| Trait | Analysis | Source of Variation | Df | MS | *F* | P-value | *σ^2^* |
| --- | --- | --- | --- | --- | --- | --- | --- |
| Paraquat  Resistance | Sexes Pooled | Sex | 1 | 5278.91 | 33.51 | <0.0001 | NA |
|  |  | *Wolbachia* | 1 | 4069.55 | 5.57 | 0.0194 | NA |
|  |  | Sex**Wolbachia* | 1 | 23.09 | 0.15 | 0.7023 | NA |
|  |  | Line(*Wolbachia)* | 165 | 730.68 | 4.64 | <0.0001 | 11.94 |
|  |  | Sex*Line(*Wolbachia)* | 165 | 157.51 | 5.13 | <0.0001 | 5.28 |
|  |  | Error | 7682 | 30.72 |  |  | 30.72 |
|  | Females | *Wolbachia* | 1 | 2352.85 | 4.38 | 0.0378 | NA |
|  |  | Line(*Wolbachia)* | 165 | 536.85 | 15.53 | <0.0001 | 20.93 |
|  |  | Error | 3841 | 34.46 |  |  | 34.46 |
|  | Males | *Wolbachia* | 1 | 1739.79 | 4.95 | 0.0274 | NA |
|  |  | Line(*Wolbachia)* | 165 | 351.35 | 13.02 | <0.0001 | 13.51 |
|  |  | Error | 3841 | 26.98 |  |  | 26.98 |
| MSB resistance | Sexes Pooled | Sex | 1 | 10131.00 | 20.87 | <0.0001 | NA |
|  |  | *Wolbachia* | 1 | 6251.84 | 2.38 | 0.0945 | NA |
|  |  | Sex**Wolbachia* | 1 | 1874.85 | 3.86 | 0.0510 | NA |
|  |  | Line(*Wolbachia*) | 165 | 2209.96 | 4.55 | <0.0001 | 35.93 |
|  |  | Sex*Line(*Wolbachia)* | 165 | 485.36 | 7.82 | <0.0001 | 17.64 |
|  |  | Error | 7682 | 62.10 |  |  | 62.10 |
|  | Females | *Wolbachia* | 1 | 639.71 | 0.40 | 0.5286 | NA |
|  |  | Line(*Wolbachia)* | 165 | 1604.04 | 22.82 | <0.0001 | 63.91 |
|  |  | Error | 3841 | 70.28 |  |  | 70.28 |
|  | Male | *Wolbachia* | 1 | 7486.99 | 6.86 | 0.0096 | NA |
|  |  | Line(*Wolbachia)* | 165 | 1091.28 | 20.24 | <0.0001 | 43.22 |
|  |  | Error | 3841 | 53.91 |  |  | 53.91 |

df: degrees of freedom; MS: Type III Mean Squares; *σ^2^*: variance component. *Phenotypic line-sex means adjusted for *Wolbachia* infection status
