# Supplementary material for: Genome-Wide Association Analysis of Oxidative Stress Resistance in Drosophila melanogaster
Source: PLoS One. 2012 Apr 4;7(4):e34745. doi: 10.1371/journal.pone.0034745 (PMC3319608; doi:10.1371/journal.pone.0034745)
Supplement: Table S3 — Analyses of variance of females and males pooled across treatments. (DOC) [file pone.0034745.s003.doc]

**Table S3. Analyses of variance of females and males pooled across treatments**

df: degrees of freedom; MS: Type III Mean Squares; *σ^2^*: variance component. *Phenotypic line-sex means adjusted for *Wolbachia* infection status

| Analysis | Source of Variation | Df | MS | *F* | *P* | *σ^2^* |
| --- | --- | --- | --- | --- | --- | --- |
| Females* | Treatment | 1 | 200205 | 281.66 | <0.0001 |  |
|  | Line | 166 | 1421.03 | 2.00 | <0.0001 | 14.80 |
|  | Line*Treatment | 166 | 710.81 | 13.57 | <0.0001 | 27.43 |
|  | Error | 7682 | 52.37 |  |  | 52.37 |
| Males* | Treatment | 1 | 461090 | 784.51 | <0.0001 |  |
|  | Line | 166 | 1026.60 | 1.75 | 0.0002 | 9.14 |
|  | Line*Treatment | 166 | 587.74 | 14.53 | <0.0001 | 22.80 |
|  | Error | 7682 | 40.45 |  |  | 40.45 |
