# Supplementary material for: Genome-Wide Association Analysis of Oxidative Stress Resistance in Drosophila melanogaster
Source: PLoS One. 2012 Apr 4;7(4):e34745. doi: 10.1371/journal.pone.0034745 (PMC3319608; doi:10.1371/journal.pone.0034745)
Supplement: Table S4 — Quantitative genetic analysis of females and males pooled across treatments. (DOC) [file pone.0034745.s004.doc]

**Table S4. Quantitative genetic analysis of females and males pooled across treatments**

*Phenotypic line-sex means adjusted for *Wolbachia* infection status

| Parameter | Symbol | Females* | Males* |
| --- | --- | --- | --- |
| Mean | *μ* | 20.45 | 21.52 |
| Genetic variance | *σ_G_^2^* | 42.23 | 31.94 |
| Genetic standard deviation | *σ_G_* | 6.50 | 5.65 |
| Environmental variance | *σ_E_^2^* | 52.37 | 40.45 |
| Environmental standard deviation | *σ_E_* | 7.24 | 6.36 |
| Phenotypic variance | *σ_P_^2^* | 94.60 | 72.39 |
| Phenotypic standard deviation | *σ_P_* | 9.73 | 8.51 |
| Heritability | *H^2^* | 0.45 | 0.44 |
| Coefficient of genetic variation | *CV_G_* | 31.78 | 26.26 |
| Coefficient of environmental variation | *CV_E_* | 35.39 | 29.55 |
| Cross-treatment genetic correlation | *r_PM_* | 0.35 | 0.29 |
