# Supplementary material for: Genome-Wide Association Analysis of Oxidative Stress Resistance in Drosophila melanogaster
Source: PLoS One. 2012 Apr 4;7(4):e34745. doi: 10.1371/journal.pone.0034745 (PMC3319608; doi:10.1371/journal.pone.0034745)
Supplement: Table S8 — Validation mutant test P-values. (DOC) [file pone.0034745.s008.doc]

**Table S8. Validation mutant test *P*-values**

|  | Paraquat | | MSB | |
| --- | --- | --- | --- | --- |
| Gene | Females | Males | Females | Males |
| *CG9650* | 0.0110 | 0.0010 | <0.0001 | <0.0001 |
| *Eip75B* | 0.5072 | 0.1745 | 0.0074 | 0.01853 |
| *ena* | 0.9955 | 0.0027 | 0.7712 | <0.0001 |
| *fog* | 0.8826 | 0.0067 | 0.2837 | 0.9880 |
| *hbn* | 0.4804 | 0.0110 | 0.2667 | 0.0189 |
| *nACRα-30D* | 0.2158 | 0.02403 | 0.4008 | 0.6173 |
| *rg* | <0.0001 | 0.0054 | 0.4585 | 0.4273 |
